# Extensive Dermatophytosis Caused by Terbinafine-Resistant *Trichophyton indotineae*, France

**DOI:** 10.3201/eid2801.210883

**Published:** 2022-01

**Authors:** Arnaud Jabet, Sophie Brun, Anne-Cecile Normand, Sebastien Imbert, Mohammad Akhoundi, Eric Dannaoui, Laeticia Audiffred, Francois Chasset, Arezki Izri, Liliane Laroche, Renaud Piarroux, Claude Bachmeyer, Christophe Hennequin, Alicia Moreno Sabater

**Affiliations:** Assistance Publique-Hôpitaux de Paris, Paris, France (A. Jabet, S. Brun, A.-C. Normand, M. Akhoundi, E. Dannaoui, L. Audiffred, F. Chasset, A. Izri, L. Laroche, R. Piarroux, C. Bachmeyer, C. Hennequin, A. Moreno Sabater);; Université Sorbonne Paris Nord, Bobigny, France (S. Brun);; Centre Hospitalier Universitaire de Bordeaux, Bordeaux, France (S. Imbert);; Université de Bordeaux, Pessac, France (S. Imbert); Université de Paris, Paris (E. Dannaoui);; Sorbonne Université, Paris (F. Chasset, R. Piarroux, C. Hennequin, A. Moreno Sabater);; Aix-Marseille Université Unité des virus émergents, Marseille, France (A. Izri).

**Keywords:** dermatophytosis, terbinafine, antimicrobial resistance, Trichophyton indotineae, ringworm, skin conditions, genotype VIII, squalene epoxidase, fungi, France, zoonoses

## Abstract

Extensive dermatophytosis caused by terbinafine-resistant *Trichophyton indotineae *harboring Phe397Leu and Leu393Ser substitutions in the squalene epoxidase enzyme was diagnosed in France. Analysis of internal transcribed spacer sequences revealed the wide spread of this species in Asia and Europe. Detection of *T. indotineae* in animals suggests their possible role as reservoirs.

In recent years, dermatologists in India have alerted the medical community to the wide spread of recalcitrant extensive dermatophytosis across the country. Clinically, extensive dermatophytosis is characterized by tinea cruris, tinea corporis, or both, of the glabrous skin (*1*). The spread of this condition is thought to be a consequence of ill-advised use of over-the-counter corticosteroid-antifungal combinations, resulting in the emergence of terbinafine-resistant *Trichophyton* strains in India (*2*). *T. mentagrophytes* and *T. interdigitale* were suspected, but the correct identity of the etiologic agent of this outbreak was debated (*3*,*4*). Genomic data showed that India terbinafine-resistant isolates form a distinct clade from *T. interdigitale* and *T. mentagrophytes* (*5*). Recently, the clinical, mycological, and molecular features of 2 highly resistant *T. interdigitale* isolates from patients from Nepal and India, harboring mutations in the squalene epoxidase (SQLE) gene, have been analyzed and identified as a new species named *T. indotineae* (*6*). 

The taxonomy of the *T. mentagrophytes*/*T. interdigitale* complex, including *T. indotineae*, has been revised based on multigene phylogeny revealing that analysis of the high-mobility group gene clearly demarcates the species, as suggested elsewhere (*5*,*7*). *T. indotineae* appears to be the primary contributor to terbinafine resistance (*8*). Migration and travel enable the spread of nonautochthonous pathogens in nonendemic areas. Terbinafine-resistant *T. indotineae* isolates have been recently identified in Europe (*9*–*11*). We present a series of extensive dermatophytosis cases in France caused by terbinafine-resistant *T. indotineae* and document the worldwide spread of *T. indotineae* using results from internal transcribed spacer (ITS) sequence-based screening.

## The Study

Ten patients in 4 hospitals in France with clinical manifestations of tinea cruris or tinea corporis were diagnosed with extensive dermatophytosis caused by *T. mentagrophytes* (Table; Appendix Figure 1). The first case was observed in 2017, and the involvement of *T. indotineae*, confirmed in 2019, revealed that the condition was probably introduced into France several years earlier. Patients were 9 men and 1 woman (mean age 30 years, range 16–53 years). All but 1 patient came from or had visited Bangladesh (Table), a commonality probably related to a 2016 increase in migrants from Bangladesh applying for asylum in France (*12*). Source of infection was difficult to determine. Patients 3 and 10 reported clinical symptoms after a journey in Bangladesh. Patients 6 and 9 reported that symptoms started before arriving in France after a stay in refugee camps in Turkey and Bangladesh. For patients 1, 4, and 8, skin lesions had appeared after living in France for 3–4 years, suggesting that human-to-human transmission could have occurred in France. All patients declared no contact with animals.

**Table:** Characteristics of extensive dermatophytosis case-patients diagnosed with *Trichophyton indotineae* in France*

Patient no.	Year	Patient country of origin	Treatments†	Clinical outcome‡	Follow up	ITS genotype§	TRB MIC,¶ μg/mL	SQLE# substitution	ITR MIC, μg/mL	VOR MIC, μg/mL	AMO MIC, μg/mL
1	2017	India	Oral and cream TRB 1 mo	Clinical cure; negative MyE	No relapse after 6 mo	ND	ND	ND	ND	ND	ND
2	2018	Bangladesh	TRB 1 mo	Improvement§ after 1 mo	Lost to follow-up	ND	ND	ND	ND	ND	ND
3	2019	Bangladesh	TRB 2 mo	Clinical cure	No relapse 1 y later	*T. indotineae*	0.06	None	0.125	0.125	0.125
4	2019	Bangladesh	TRB 3 mo, GRS 3 mo, ECZ 3 mo	No improvement after 9 mo; positive MyE	Lost to follow-up	*T. indotineae*	2	Leu393Ser	ND	ND	ND
5	2020	Bangladesh	TRB 2 mo	No improvement after 2 mo; positive MyE	Lost to follow-up	*T. indotineae*	>8	Phe397Leu	0.06	0.06	0.06
6	2020	Myanmar	CCL 1 mo	Clinical cure	No relapse 1 y later	*T. indotineae*	0.06	Ala448Thr	0.125	0.125	0.06
7	2020	Bangladesh	TRB 3 wk, BFN 3 wk	Improvement after 6 wk	Relapse 2 mo later	*T. indotineae*	2	Leu393Ser	0.016	0.03	0.06
8	2020	Bangladesh	OMC 1 mo, MCN 1 mo	Improvement after 2 mo	Lost to follow-up	ND	ND	ND	ND	ND	ND
9	2021	Bangladesh	TRB 2 mo	Improvement after 1 mo; negative MyE	Lost to follow-up	*T. indotineae*	0.06	None	0.06	0.06	0.125
10	2021	Bangladesh	TRB 6 mo, GRS 6 mo	No improvement after 1 y; positive MyE	ITR 2 mo improvement	*T. indotineae*	2	Phe397Leu Ala448Thr	0.25	0.5	0.01

*AMO, amorolfine; BFN, bifonazole; CCL, ciclopiroxolamine; ECZ, econazole; GRS, griseofulvin; ITR, itraconazole; ITS, internal transcribed spacer; MCN, miconazole; MyE, mycologic exam; ; ND, not determined; OMC, omoconazole; SQLE, squalene epoxidase enzyme; TRB, terbinafine; VOR, voriconazole. †Treatments: oral TRB (250 mg/d); oral GRS (1 g/d); 1% ECZ cream; 1% CCL cream; 1% BFN cream; 1% OMC cream; 2% MCN cream; oral ITR (400 mg/d). ‡Clinical cure was recorded when skin lesions disappeared after treatment; clinical improvement was recorded when patient reported a reduction of clinical symptoms (itching and inflammatory lesions). §*T. indotineae* ITS sequencing was performed using primers (Appendix Table). Registered under GenBank accession nos. MW959755–60. ¶TBR resistance was defined by a MIC_50_ >0.25 µg/mL (*8*). #For SQLE sequencing, the amplified fragment was cut in 2 using a total of 4 primers because it was >1,000 nt long, (Appendix Table). Registered under GenBank accession no. MZ318454–9.

Eight out of 10 patients received oral treatment with terbinafine. Clinical cure or treatment failure were difficult to evaluate because most patients could not be reached for follow-up. We observed clinical cure in 2/8 patients (patients 1 and 3) and clinical improvement in 2/8 patients (patients 2 and 9). However, we observed no clinical improvement or relapse in 4/8 patients (patients 4, 5, 7, and 10). Patient 4 received a second round of oral griseofulvin treatment during the 3 months after initial treatment, and patient 10 received a second round of oral terbinafine for 3 months followed by griseofulvin treatment over 6 months, without clear clinical improvement for either. Finally, we observed clinical improvement in patient 10 after 1 month of a 2-month treatment with itraconazole (400 mg/d). Indeed, limited effectiveness of 4 oral antifungals (fluconazole, griseofulvin, itraconazole, and terbinafine) has been described in India, but itraconazole has shown better efficacy than the others (*13*). Increasing terbinafine exposure through higher doses or longer durations has been proposed to overcome treatment failure and could protect azole-based antifungal drugs from developing resistance (*14*).

Seven isolates initially diagnosed as *T. mentagrophytes* by microscopy, mass spectrometry, and clinical localization were available for further characterization (Table). ITS region sequencing revealed that all isolates were *T. indotineae*. Three out of 6 isolates grew on a solid terbinafine-containing medium (Appendix Figure 2). We confirmed terbinafine resistance by the EUCAST (https://eucast.org) method (*15*) with MICs of 2 or >8 μg/mL (Table). Sequencing the SQLE gene revealed that 4 resistant isolates contained Leu393Ser, Phe397Leu, or Phe397Leu/Ala448Thr substitutions. Three terbinafine-susceptible isolates exhibited no substitutions or an Ala448Thr substitution alone. We determined low MICs for itraconazole, voriconazole, and amorolfine for 6 isolates (Table).

Using an ITS sequence-based screening of sequences stored in GenBank and a review of literature up through March 2021 (Appendix Table 2), we investigated the epidemiologic characteristics of *T. indotineae*. Information was available about the origin of the infection for 526/537 sequences found. Human-to-human transmission was predominant because 98.8% of the sequences identified were of human origin; however, 6 sequences indicated an animal origin. Two sequences detected during a survey that included 760 calves came from Egypt, 1 detected in an infected dog came from India, and 3 came from Poland but with no specific animal host indicated. These results suggest that animals can be reservoirs of *T. indotineae* and zoonotic transmission must be considered.

**Figure 1:**
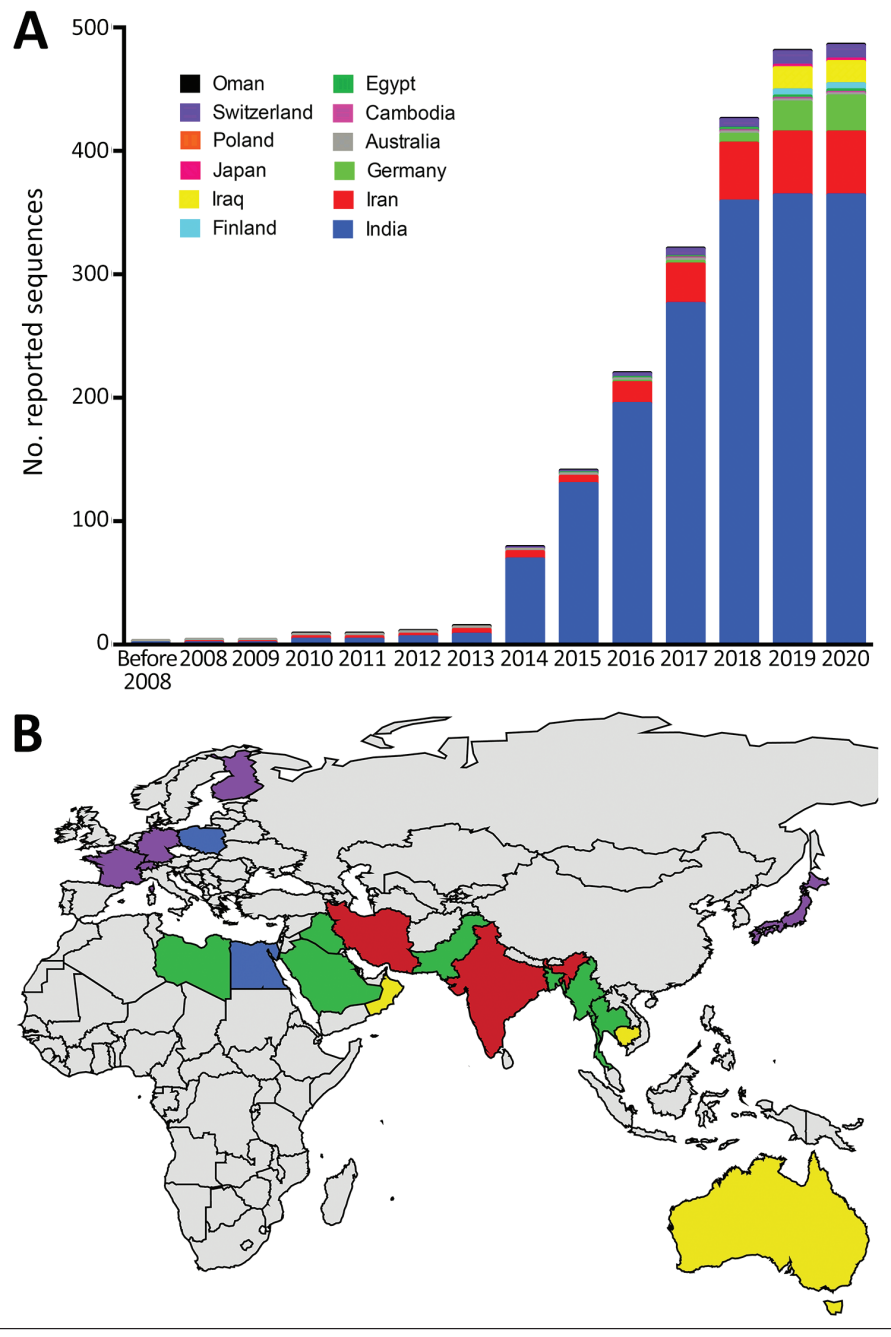
Analysis of dynamic and geographic distribution of *Trichophyton indotineae* reported sequences from France (this study) and reference sequences from GenBank for 2004–2021 A) Cumulative curves of 486 published sequences; B) geographic distribution of 537 published sequences. Red, countries with reported endemic cases; purple, countries with imported cases (but rare cases of endemic transmission cannot be ruled out); green, probable country sources of imported cases; yellow, countries with reported sporadic human cases without additional available information (also identified in Poland); blue, countries with *T. indotineae* sequences reported in animal infections (also reported in India). World map was created using JMP Pro 15.2.0 (https://www.jmp.com). For internal transcribed spacer sequence-based screening, we retrieved ITS1-5.8S-ITS2 sequences *T. interdigitale*, *T. mentagrophytes*, *T. indotineae* and also *Anthroderma benhamiae*, *A. simii*, *A. vanbreuseghemii*, *T. benhamiae*, *T. bullosum*, *T. concentricum*, *T. equinum*, *T. erinacei*, *T. quinckeanum*, *T. simii*, *T. schoenleinii*, *T. tonsurans*, and *T. verrucosum.* For sequences matching *T. indotineae* (internal transcribed spacer reference sequence JN133999), we searched associated literature on PubMed Central (https://www.ncbi.nlm.nih.gov/pmc).

We obtained geographic information for all 537 sequences and temporal information for 486 sequences. Our study revealed that *T. indotineae* was present in India, Australia, Iran, and Oman during 2004–2013 (Figure 1, panel A). After 2014, a substantial increase in reported cases was observed, related to the outbreak in India. Since 2019, the number of reported *T. indotineae* cases has increased in Europe, confirming its spread. Currently, 76% of the known sequences have been identified in India, 12.8% in the Middle East, 9.6% in Europe, and 1.1% in other countries (Figure 1, panel B). Cases reported in Europe are supposed to have been introduced by migrants or travelers from India, Bangladesh, Pakistan, Bahrain, Libya, Saudi Arabia, or Thailand, suggesting the presence of *T. indotineae* in those countries. The cases imported from Bangladesh that were reported in France, together with those reported in Germany (*9*), suggest that *T. indotineae* transmission could be endemic in Bangladesh. Results obtained in this study were limited to data obtained from sources available in GenBank; *T. indotineae* distribution is probably greater than what is documented here.

**Figure 2:**
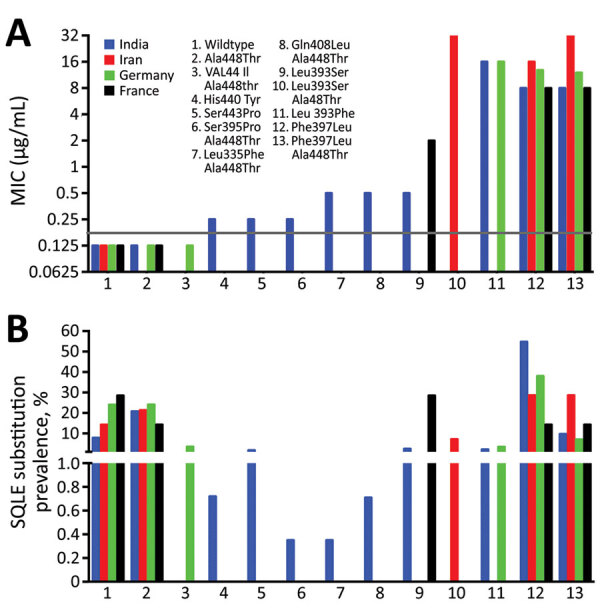
*Trichophyton indotineae* susceptibility to terbinafine reported from 4 countries. A) Relationships between terbinafine MIC and codon changes reported in isolates from different countries. Grey line shows terbinafine susceptibility threshold of 0.2 μg/mL. Available MICs were determined using the Clinical Laboratory and Standards Institute (https://clsi.org) or EUCAST (https://eucast.org) methods. Data show mean values. B) Prevalence of substitution points in the gene encoding SQLE. Sources shown in the Appendix. SQLE, squalene epoxidase enzyme.

The epidemiology of terbinafine-resistant *T. indotineae* isolates was difficult to assess because studies recording available molecular analysis and in vitro antifungal susceptibility testing were scarce. In India, 71.3% (n = 279) of reported isolates were resistant to terbinafine, but in Iran, 71.8% (n = 32) were susceptible. Of the isolates from this study, 50% (n = 29) from Germany and 57.1% (n = 7) from France were resistant. Isolates from India showed 11 different single or combined missense mutations of the SQLE gene with a large range of terbinafine MICs (Figure 2). Of note, Phe397Leu and Leu393Phe substitutions, associated with terbinafine resistance, were predominant in isolates from India and Germany, probably related to population movements between the 2 countries. Leu393Ser substitution was predominant in France in the isolates from Bangladesh.

## Conclusions

The medical community and organizations receiving migrants and travelers should be aware that extensive dermatophytosis linked to terbinafine-resistant *T. indotineae* has reached France. Efficient systems to promptly identify terbinafine-resistant *T. indotineae* isolates must be implemented to halt the progression of this pathogen throughout Europe. 

Appendix Additional information on dermatophytosis caused by terbinafine-resistant *Trichophyton indotineae.*

## References

[1] Dogra S, Uprety S. The menace of chronic and recurrent dermatophytosis in India: Is the problem deeper than we perceive? Indian Dermatol Online J. 2016;7:73–6. 10.4103/2229-5178.178100 27057485 PMC4804598

[2] Bishnoi A, Vinay K, Dogra S. Emergence of recalcitrant dermatophytosis in India. [Comment in Lancet Infect Dis. 2018;18:718–9.]. Lancet Infect Dis. 2018;18:250–1. 10.1016/S1473-3099(18)30079-3 29485088

[3] Nenoff P, Verma SB, Uhrlaß S, Burmester A, Gräser Y. A clarion call for preventing taxonomical errors of dermatophytes using the example of the novel *Trichophyton mentagrophytes* genotype VIII uniformly isolated in the Indian epidemic of superficial dermatophytosis. Mycoses. 2019;62:6–10. 10.1111/myc.12848 30187579

[4] Chowdhary A, Singh A, Singh PK, Khurana A, Meis JF. Perspectives on misidentification of *Trichophyton interdigitale*/*Trichophyton mentagrophytes* using internal transcribed spacer region sequencing: Urgent need to update the sequence database. Mycoses. 2019;62:11–5. 10.1111/myc.12865 30367553

[5] Singh A, Masih A, Monroy-Nieto J, Singh PK, Bowers J, Travis J, et al. A unique multidrug-resistant clonal *Trichophyton* population distinct from *Trichophyton mentagrophytes*/*Trichophyton interdigitale* complex causing an ongoing alarming dermatophytosis outbreak in India: Genomic insights and resistance profile. Fungal Genet Biol. 2019;133:103266. 10.1016/j.fgb.2019.103266 31491507

[6] Kano R, Kimura U, Kakurai M, Hiruma J, Kamata H, Suga Y, et al. *Trichophyton indotineae* sp. nov.: a new highly terbinafine-resistant anthropophilic dermatophyte species. Mycopathologia. 2020;185:947–58. 10.1007/s11046-020-00455-8 32449054

[7] Tang C, Kong X, Ahmed SA, Thakur R, Chowdhary A, Nenoff P, et al. Taxonomy of the *Trichophyton mentagrophytes*/*T. interdigitale* species complex harboring the highly virulent, multiresistant genotype *T. indotineae.* Mycopathologia. 2021;186:315–26. 10.1007/s11046-021-00544-2 33847867 PMC8249266

[8] Ebert A, Monod M, Salamin K, Burmester A, Uhrlaß S, Wiegand C, et al. Alarming India-wide phenomenon of antifungal resistance in dermatophytes: A multicentre study. Mycoses. 2020;63:717–28. 10.1111/myc.13091 32301159

[9] Nenoff P, Verma SB, Ebert A, Süß A, Fischer E, Auerswald E, et al. Spread of terbinafine-resistant *Trichophyton mentagrophytes* type VIII (India) in Germany—“the tip of the iceberg? J Fungi (Basel). 2020;6:207. 10.3390/jof6040207 33027904 PMC7712673

[10] Klinger M, Theiler M, Bosshard PP. Epidemiological and clinical aspects of *Trichophyton mentagrophytes*/*Trichophyton interdigitale* infections in the Zurich area: a retrospective study using genotyping. J Eur Acad Dermatol Venereol. 2021;35:1017–25. 10.1111/jdv.17106 33411941

[11] Saunte DML, Pereiro-Ferreirós M, Rodríguez-Cerdeira C, Sergeev AY, Arabatzis M, Prohić A, et al. Emerging antifungal treatment failure of dermatophytosis in Europe: take care or it may become endemic. J Eur Acad Dermatol Venereol. 2021;35:1582–6. 10.1111/jdv.17241 33768571

[12] Office Français de protection des réfugiés et apatrides. 2016 activity report [cited on 19 Apr 2021]. https://ofpra.gouv.fr/sites/default/files/atoms/files/rapport_dactivite_ofpra_2016_1.pdf

[13] Singh S, Chandra U, Anchan VN, Verma P, Tilak R. Limited effectiveness of four oral antifungal drugs (fluconazole, griseofulvin, itraconazole and terbinafine) in the current epidemic of altered dermatophytosis in India: results of a randomized pragmatic trial. Br J Dermatol. 2020;183:840–6. 10.1111/bjd.19146 32538466

[14] Khurana A, Masih A, Chowdhary A, Sardana K, Borker S, Gupta A, et al. Correlation of *in vitro* susceptibility based on MICs and squalene epoxidase mutations with clinical response to terbinafine in patients with tinea corporis/cruris. Antimicrob Agents Chemother. 2018;62:e01038–18. 10.1128/AAC.01038-18 30275090 PMC6256768

[15] Arendrup MC, Jørgensen KM, Guinea J, Lagrou K, Chryssanthou E, Hayette MP, et al. Multicentre validation of a EUCAST method for the antifungal susceptibility testing of microconidia-forming dermatophytes. J Antimicrob Chemother. 2020;75:1807–19. 10.1093/jac/dkaa111 32303059

