# Supplementary material for: Extensive Dermatophytosis Caused by Terbinafine-Resistant Trichophyton indotineae, France
Source: Emerg Infect Dis. 2022 Jan;28(1):229–33. doi: 10.3201/eid2801.210883 (PMC8714191; doi:10.3201/eid2801.210883)
Supplement: Appendix — Additional information on dermatophytosis caused by terbinafine-resistant Trichophyton indotineae. [file 21-0883-Techapp-s1.pdf]

Article DOI: https://doi.org/10.3201/eid2801.210883

Extensive Dermatophytosis Caused by Terbinafine-Resistant *Trichophyton indotineae*, France

Appendix

Appendix Table. PCR primers used in this study and annealing temperatures*

DNA portion	Primer	Sequence	Annealing temperature
ITS	ITS1	TCCGTAGGTGAACCTGCGG	55°C
	ITS3	GCATCGATGAAGAACGCAGC	
	ITS4c	TCCTCCGCTTATTGATATGC	
Squalene epoxidase	Erg1_2-F	CCAGACTGATGGCAAGCAAGA	60°C (used only for sequencing)
	Erg1–2_R	ATAAGCTCCAGGCCCCAGAA	
	TrSQLE-F1	ATGGTTGTAGAGGCTCCTCCC	
	TrSQLE-R1	CTAGCTTTGAAGTTCGGCAAA	

* Primers were previously described by Yamada et al. (*1*). ITS, internal transcribed spacer.

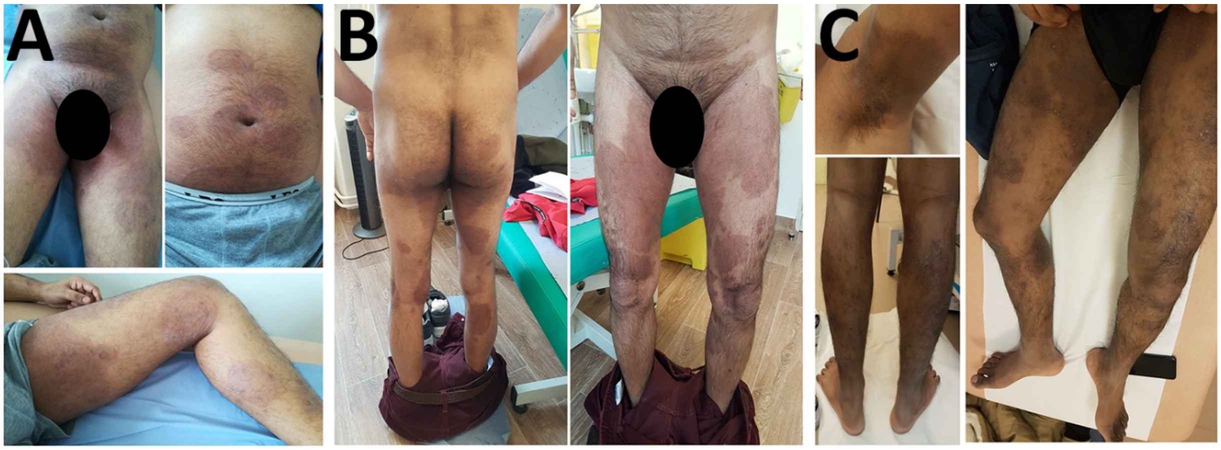

Appendix Figure 1. Clinical presentation of extensive dermatophytosis diagnosed in France. A–B) The various lesions consisted in multiple and large erythematous or brown, round to oval macular lesions on the abdomen, legs, arms and inguinal folds, slightly scaly, with a raised inflammatory edge. Patients reported peripheral spreading but no central clearing. C) An atypical clinical presentation was observed in one patient (patient 9) with large and brown annular macular lesions and no inflammatory edge.

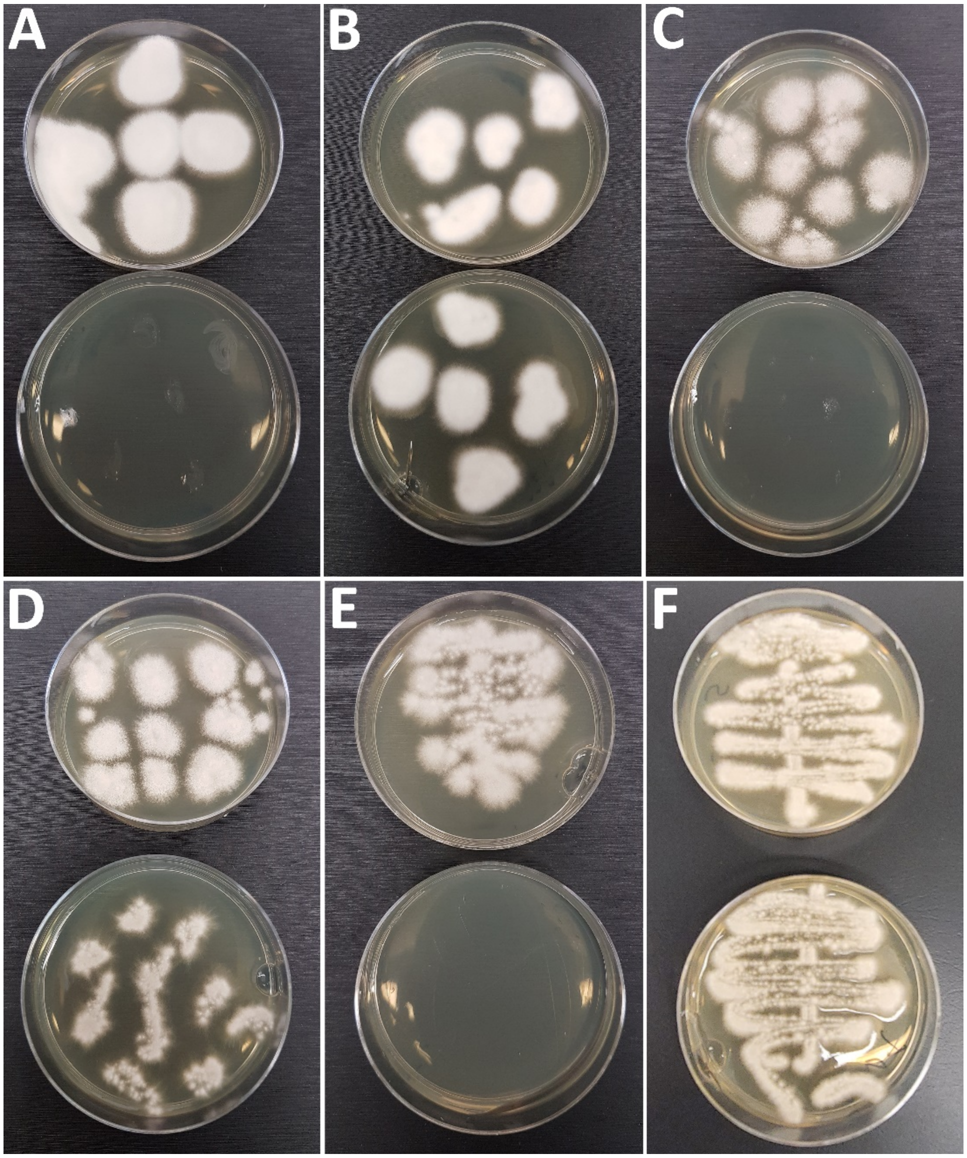

Appendix Figure 2. Terbinafine susceptibility screening using the solid plate method. Photos show antifungal susceptibility to terbinafine determined using the growth method involving Sabouraud-chloramphenicol-cycloheximide medium (SCC) with or without terbinafine at 0.2 µg/mL (lower and uppers photos respectively). A–F) Patients 3, 5, 6, 7, 9, 10, respectively. Terbinafine susceptibility using this method could be determined for 6 isolates (loss of viability for isolate 4). Isolates from patients 5, 7,10 were able to grow in terbinafine-containing solid medium.

Reference

1. Yamada T, Maeda M, Alshahni MM, Tanaka R, Yaguchi T, Bontems O, et al. Terbinafine resistance of *Trichophyton* clinical isolates caused by specific point mutations in the squalene epoxidase gene. Antimicrob Agents Chemother. 2017;61:e00115-17.

Additional Sources

1. Tartor YH, El-Neshwy WM, Merwad AMA, Abo El-Maati MF, Mohamed RE, Dahshan HM, et al. Ringworm in calves: risk factors, improved molecular diagnosis, and therapeutic efficacy of an Aloe vera gel extract. BMC Vet Res. 2020;16:421. PubMed https://doi.org/10.1186/s12917-020-02616-9

2. Nenoff P, Verma SB, Ebert A, Süß A, Fischer E, Auerswald E, et al. Spread of terbinafine-resistant *Trichophyton mentagrophytes* type VIII (India) in Germany—"the tip of the iceberg? J Fungi (Basel). 2020;6:207. PubMed https://doi.org/10.3390/jof6040207

3. Burmester A, Hipler UC, Hensche R, Elsner P, Wiegand C. Point mutations in the squalene epoxidase gene of Indian ITS genotype VIII *T. mentagrophytes* identified after DNA isolation from infected scales. Med Mycol Case Rep. 2019;26:23–4. PubMed https://doi.org/10.1016/j.mmcr.2019.09.001

4. Kong F, Tong Z, Chen X, Sorrell T, Wang B, Wu Q, et al. Rapid identification and differentiation of *Trichophyton* species, based on sequence polymorphisms of the ribosomal internal transcribed spacer regions, by rolling-circle amplification. J Clin Microbiol. 2008;46:1192–9. PubMed https://doi.org/10.1128/JCM.02235-07

5. Gangneux J-P, Lortholary O, Cornely OA, Pagano L. 9th trends in medical mycology held on 11–14 October 2019, Nice, France, organized under the auspices of EORTC-IDG and ECMM. J Fungi (Basel). 2019;5:95. PubMed https://doi.org/10.3390/jof5040095

6. Singh A, Masih A, Monroy-Nieto J, Singh PK, Bowers J, Travis J, et al. A unique multidrug-resistant clonal *Trichophyton* population distinct from *Trichophyton mentagrophytes*/*Trichophyton interdigitale* complex causing an ongoing alarming dermatophytosis outbreak in India: Genomic insights and resistance profile. Fungal Genet Biol. 2019;133:103266. PubMed https://doi.org/10.1016/j.fgb.2019.103266

7. Ebert A, Monod M, Salamin K, Burmester A, Uhrlaß S, Wiegand C, et al. Alarming India-wide phenomenon of antifungal resistance in dermatophytes: A multicentre study. Mycoses. 2020;63:717–28. PubMed https://doi.org/10.1111/myc.13091

8. Chowdappa A, Patil S, Nagmoti MB. Isolation and molecular characterization of *Trichophyton rubrum* from Siddi tribal community residing in North Karnataka Region, India. J Evol Med Dent Sci. 2020;9:2208–11. https://doi.org/10.14260/jemds/2020/480

9. Burmester A, Hipler UC, Uhrlaß S, Nenoff P, Singal A, Verma SB, et al. Indian *Trichophyton mentagrophytes* squalene epoxidase erg1 double mutants show high proportion of combined fluconazole and terbinafine resistance. [Online ahead of print.]. Mycoses. 2020;63:1175–80. PubMed https://doi.org/10.1111/myc.13150

10. Shaw D, Singh S, Dogra S, Jayaraman J, Bhat R, Panda S, et al. MIC and upper limit of wild-type distribution for 13 antifungal agents against a *Trichophyton mentagrophytes-Trichophyton interdigitale* complex of Indian origin. Antimicrob Agents Chemother. 2020;64:e01964–19. PubMed https://doi.org/10.1128/AAC.01964-19

11. Ramaraj V, Vijayaraman RS, Hemanth V, Rangarajan S, Kindo AJ. Molecular strain typing of *Trichophyton mentagrophytes* (*T. mentagrophytes* var. *interdigitale*) using non-transcribed spacer region as a molecular marker. Indian J Med Res. 2017;146:636–41. PubMed

12. Nenoff P, Verma SB, Vasani R, Burmester A, Hipler UC, Wittig F, et al. The current Indian epidemic of superficial dermatophytosis due to *Trichophyton mentagrophytes*-A molecular study. Mycoses. 2019;62:336–56. PubMed https://doi.org/10.1111/myc.12878

13. Shankarnarayan SA, Shaw D, Sharma A, Chakrabarti A, Dogra S, Kumaran MS, et al. Rapid detection of terbinafine resistance in *Trichophyton* species by Amplified refractory mutation system-polymerase chain reaction. Sci Rep. 2020;10:1297. PubMed https://doi.org/10.1038/s41598-020-58187-0

14. Pchelin IM, Azarov DV, Churina MA, Scherbak SG, Apalko SV, Vasilyeva NV, et al. Species boundaries in the *Trichophyton mentagrophytes* / *T. interdigitale* species complex. Med Mycol. 2019;57:781–9. PubMed https://doi.org/10.1093/mmy/myy115

15. Kawasaki M, Anzawa K, Wakasa A, Takeda K, Tanabe H, Mochizuki T, et al. Different genes can result in different phylogenetic relationships in *trichophyton* species. Nippon Ishinkin Gakkai Zasshi. 2008;49:311–8. PubMed https://doi.org/10.3314/jjmm.49.311

16. Rezaei-Matehkolaei A, Rafiei A, Makimura K, Gräser Y, Gharghani M, Sadeghi-Nejad B. Epidemiological aspects of dermatophytosis in Khuzestan, southwestern Iran, an update. Mycopathologia. 2016;181:547–53. PubMed https://doi.org/10.1007/s11046-016-9990-x

17. Rezaei-Matehkolaei A, Makimura K, de Hoog S, Shidfar MR, Zaini F, Eshraghian M, et al. Molecular epidemiology of dermatophytosis in Tehran, Iran, a clinical and microbial survey. Med Mycol. 2013;51:203–7. PubMed https://doi.org/10.3109/13693786.2012.686124

18. Zareshahrabadi Z, Totonchi A, Rezaei-Matehkolaei A, Ilkit M, Ghahartars M, Arastehfar A, et al. Molecular identification and antifungal susceptibility among clinical isolates of dermatophytes in Shiraz, Iran (2017-2019). Mycoses. 2021;64:385–93. PubMed https://doi.org/10.1111/myc.13226

19. Taghipour S, Pchelin IM, Zarei Mahmoudabadi A, Ansari S, Katiraee F, Rafiei A, et al. Trichophyton mentagrophytes and T interdigitale genotypes are associated with particular geographic areas and clinical manifestations. Mycoses. 2019;62:1084–91. PubMed https://doi.org/10.1111/myc.12993

20. Kano R, Kimura U, Kakurai M, Hiruma J, Kamata H, Suga Y, et al. *Trichophyton indotineae* sp. nov.: a new highly terbinafine-resistant anthropophilic dermatophyte species. Mycopathologia. 2020;185:947–58. PubMed https://doi.org/10.1007/s11046-020-00455-8

21. Klinger M, Theiler M, Bosshard PP. Epidemiological and clinical aspects of *Trichophyton mentagrophytes*/*Trichophyton interdigitale* infections in the Zurich area: a retrospective study using genotyping. J Eur Acad Dermatol Venereol. 2021;35:1017–25. PubMed https://doi.org/10.1111/jdv.17106

22. Fattahi A, Shirvani F, Ayatollahi A, Rezaei-Matehkolaei A, Badali H, Lotfali E, et al. Multidrug-resistant *Trichophyton mentagrophytes* genotype VIII in an Iranian family with generalized dermatophytosis: report of four cases and review of literature. Int J Dermatol. 2021;60:686–92. PubMed https://doi.org/10.1111/ijd.15226

23. Taghipour S, Shamsizadeh F, Pchelin IM, Rezaei-Matehhkolaei A, Zarei Mahmoudabadi A, Valadan R, et al. Emergence of terbinafine resistant *Trichophyton mentagrophytes* in Iran, harboring mutations in the squalene epoxidase (SQLE) gene. Infect Drug Resist. 2020;13:845–50. PubMed https://doi.org/10.2147/IDR.S246025
